# A retrospective, pooled data analysis of the safety of pegaptanib sodium in the treatment of age-related macular degeneration in subjects with or without diabetes mellitus

**DOI:** 10.1186/1471-2415-12-37

**Published:** 2012-08-08

**Authors:** Theresa Dombi, Kenneth K Kwok, Marla B Sultan

**Affiliations:** 1 Pfizer Inc, Groton, CT, USA; 2 Pfizer Inc, New York, NY, USA; 3 The New York Eye and Ear Infirmary, New York, NY, USA

## Abstract

**Background:**

To evaluate the safety of pegaptanib sodium 0.3 mg intravitreal injection in the treatment of neovascular age-related macular degeneration in subjects with or without diabetes mellitus.

**Methods:**

A pooled, retrospective, analysis was conducted of data from 9 sponsor-administered, randomized, open-label trials. Subjects who received pegaptanib by randomization or change in dose assignment, crossover design, or protocol amendment, were included. Reports of endophthalmitis, increased intraocular pressure, retinal injury, intraocular hemorrhage, traumatic cataract, hypersensitivity reactions, stroke, myocardial infarction, and other arterial thromboembolic events defined by the Antiplatelet Trialists’ Collaboration were identified by Medical Dictionary for Regulatory Activities preferred terms. Adverse events were summarized from the first injection to 42 days after the last injection. The incidence of adverse events was stratified by the presence/absence of diabetes.

**Results:**

Of 1,586 subjects enrolled, 165 (10.4%) had a history of diabetes mellitus and 1,421 (89.6%) did not. The 2 populations were similar at baseline. Based on the comparison of prespecified ocular, hypersensitivity, and Antiplatelet Trialists’ Collaboration event terms, the safety review did not identify any notable differences between the 2 populations.

**Conclusions:**

This retrospective analysis found no increased safety risk resulting from treatment with pegaptanib 0.3 mg in individuals with neovascular age-related macular degeneration and concomitant diabetes mellitus.

## Background

Inflammation is thought to play an important role in the pathophysiology of diabetic retinopathy (DR) [1-3] with elevated levels of vascular endothelial growth factor (VEGF) being a key mediator [4]. The off-label intravitreal use of VEGF antagonists (with the exception of ranibizumab, which is currently approved in Europe) in the treatment of DR and diabetic macular edema (DME) appears to result in vision gains and reductions in central macular thickness [5,6]. Pegaptanib sodium is a selective VEGF antagonist first approved in the United States for the treatment of age-related macular degeneration (AMD) in December 2005. In retrospective studies of pegaptanib sodium, significant reductions in central macular thickness and changes in visual acuity were reported in subjects with DME compared to baseline [7] and compared to laser alone [8]. Intravitreal pegaptanib also has been used to delay or abrogate the need for vitrectomy for recurrent and nonclearing vitreous hemorrhage in proliferative DR in a consecutive case series [9].

The diabetic population is at an increased risk of vascular complications, including myocardial infarction [10], stroke [11-14], coronary heart disease [14,15], and peripheral artery disease [16]. Although pegaptanib has an established long-term safety record in subjects with neovascular AMD [17,18] the question arises as to whether or not the intraocular administration of anti-VEGF agents in the diabetic population poses an additional systemic risk. Because there is a lack of large, comparative studies examining the systemic effects of anti-VEGF therapies, understanding the implications of administering these agents in individuals with diabetes is important information for the clinician. This retrospective analysis was performed to evaluate the safety of pegaptanib in the subset of patients with diabetes who were receiving pegaptanib for AMD (data on file, Pfizer Inc) [19,20].

## Methods

This pooled retrospective analysis evaluated the occurrence of prespecified adverse events in subjects with or without diabetes mellitus (type 1 and type 2) who were treated with pegaptanib sodium injection (Macugen, Eyetech Inc) for AMD. All subjects who received study treatment with pegaptanib 0.3 mg by intravitreal injection in the Phase II through Phase IV clinical trials by way of randomization assignment, crossover design, protocol amendment, or change in dose assignment were included in the analysis (Table 1). These studies were conducted in full conformance with the principles of the Declaration of Helsinki or with the laws of the country in which the research was conducted, whichever afforded the greater protection to the study participant. Institutional Review Board or Ethics Committee approval was obtained from each clinical center and signed informed consent was obtained from all study participants.

**Table 1:** Clinical studies included in the analysis in which subjects received 0.3 mg pegaptanib

**Study**	**Duration of Treatment**	**Treatment Groups**	**Pegaptanib sodium 0.3 mg**		**Sham**	**Reference**
			**Subjects**			
			**With Diabetes**	**Without Diabetes**	**With Diabetes**	
			**N = 165**	**N = 1421**	**N = 26**	
NCT00321997 (EOP1003; randomized)	Up to 5 years	Pegaptanib 0.3, 1, 3 mg; sham	22 (13.3%)	208 (14.6%)	7 (26.9%)	[15]
NCT00021736 (EOP1004; randomized)	Up to 5 years	Pegaptanib 0.3, 1, 3 mg; sham	20 (12.1%)	182 (12.8%)	14 (53.8%)	[17]
NCT00215670 (EOP1006; randomized)	Up to 2 years	Pegaptanib 0.3, 1, 3 mg	1 (0.6%)	41 (2.9%)	0 (0%)	[29]
NCT00087763 (EOP1009; randomized)	1 year; with no treatment weeks 12 to 24	Pegaptanib 0.3, 1 mg; sham	4 (2.4%)	60 (4.2%)	5 (19.2%)	*
NCT00088192 (EOP1010; open-label)	Up to 3 years	Pegaptanib 0.3 mg	67 (40.6%)	370 (26.0%)	0 (0%)	[30]
NCT00134667** EOP1012; randomized)	Up to 2 years	Pegaptanib 0.3 mg; pegaptanib 0.3 mg + PDT	16 (9.7%)	149 (10.5%)	0 (0%)	
NCT00150202/ NCT00239928 (A5751010/ A5751015; open-label)	Up to 4 years	Pegaptanib 0.3, 1 mg	7 (4.2%)	72 (5.1%)	0 (0%)	[30]***
NCT00324116 (A5751016; open-label)	1 year	Pegaptanib 0.3 mg	11 (6.7%)	70 (4.9%)	0 (0%)	†
NCT00327470 (A5751017; open-label)	Up to 2 years	Pegaptanib 0.3 mg	17 (10.3%)	269 (18.9%)	0 (0%)	†

PDT: photodynamic therapy with verteporfin.

*Unpublished observation ([by author or pharma, year]).

**Unpublished results; trial terminated.

***Study A5751015 was an extension of study A5751010 and were considered as a single study for this manuscript.

^†^Unpublished observations (Estephan M, Troy S, Starita C, for the French Macugen in Early Onset CNV Study Group presented at European Society of Retina Specialists - 10th EURETINA Congress. Paris, France; 2010: Poster 1016).

In studies where subjects received 0.3 mg doses and/or other doses (sham or pegaptanib 1 mg or 3 mg), only the period when they received the 0.3 mg dose was included for compiling data on treatment exposure, adverse events, concomitant medications and discontinuations. The treatment-exposure period was defined as the time from the first pegaptanib 0.3 mg injection to 42 days after the last injection.

Standard summary tables filtered by the prespecified treatment-emergent adverse event MedDRA [Medical Dictionary for Regulatory Activities, version 12.1] preferred terms (PTs) (Table 2) were prepared. These included endophthalmitis, increased intraocular pressure (IOP), retinal injury, intraocular hemorrhage, traumatic cataract, hypersensitivity reactions, stroke, myocardial infarction, and other cardiovascular and cerebrovascular thromboembolic events [21,22]. The ocular and nonocular (hypersensitivity) terms were those used to identify the ocular and hypersensitivity risks for pegaptanib in the risk management plan and for which the sponsor conducts regular pharmacovigilance monitoring. The cardiovascular, cerebrovascular and peripheral vascular arterial thromboembolic events listed were selected based on Antiplatelet Trialists’ Collaboration (APTC) criteria [21,22] and are the terms followed as part of routine pharmacovigilance by the sponsor. In order to detect whether the clinical database contained any of the prespecified treatment-emergent PTs, each PT was divided into a separate text string for extensive search purposes so that if a PT recorded in the clinical database contained any of the text strings, it was considered a match. Any unrelated terms were removed after the first-round review.

**Table 2:** Prespecified adverse event preferred terms, MedDRA version 12.1


**Ocular**	**Nonocular**
Endophthalmitis	Drug hypersensitivity
Panophthalmitis	Hypersensitivity
Intraocular pressure, increased	Hypersensitivity NOS (v. 5.1)
Ocular hypertension	Anaphylactic shock
Glaucoma	Anaphylactic reaction
Glaucoma NOS (v. 5.1)	Anaphylactoid shock
Glaucoma, traumatic	Anaphylactoid reaction
Retinal injury	Angioedema
Retinal tear	Angioneurotic edema (v. 5.1)
Retinal detachment	Blepharitis, allergic
Retinal hemorrhage	Dermatitis, contact
Retinal toxicity	Dermatitis, allergic
Detachment of retinal pigment epithelium	Toxic skin eruption
Retinal pigment epitheliopathy (v. 5.1)	Toxic epidermal necrolysis
Eye hemorrhage	Lyell syndrome (v. 5.1)
Eye hemorrhage NOS (v. 5.1)	Drug eruption
Vitreous hemorrhage	Dermatitis medicamentosa (v. 5.1)
Cataract traumatic	Erythema
Erythema multiforme	Urticaria
Tongue edema	Stevens-Johnson syndrome
Pharyngeal edema	Stevens-Johnson syndrome (v. 5.1)
Laryngeal edema	Rash
Latex allergy	Rash NOS (v. 5.1)
Paresthesia oral	Skin reaction
Paraesthesia mucosal	Acute generalized exanthematous pustulosis
Paraesthesia mucosal NOS (v. 5.1)	Drug rash with eosinophilia and systemic symptoms
**Antiplatelet Trialists’ Collaboration Events**	
**Cardiovascular**	**Cerebrovascular**
Acute myocardial infarction	Brain stem infarction
Cardiac arrest	Cerebrovascular accident
Cardiac failure congestive	Lacunar infarction
Myocardial infarction	Cerebral hemorrhage
Retroperitoneal hemorrhage	Transient ischemic attack
Sudden death	Peripheral vascular thrombosis
Silent myocardial infarction	Pulmonary embolism
Coronary artery stenosis	Deep vein thrombosis
Acute coronary syndrome	Thrombosis
Angina pectoris	Ischemia
Angina unstable	Ischemia NOS (v. 5.1)

MedDRA: Medical Dictionary for Regulatory Activities.

NOS: not otherwise specified.

V: version.

The frequency of prespecified treatment-emergent event terms as well as other safety data reported by the pooled AMD subjects with a medical history of diabetes mellitus were compared with the corresponding data reported by the pooled AMD subjects without a medical history of diabetes mellitus. Events were categorized by severity (mild, moderate, severe); in cases where the subject experienced the same event more than once, the worst severity was presented.

Because sham injections were administered to subjects with diabetes in 3 of the pooled studies, an additional analysis of APTC events reported in this population was conducted for comparison to pegaptanib-treated subjects in the primary analysis. Events occurring in subjects who received both pegaptanib and sham at different time points in the study were counted twice if the event was reported during each time period.

## Results

### Subject disposition and demographics

Nine sponsor-administered clinical studies, both randomized and open-label of 1 to 5 years’ duration, in which subjects received pegaptanib 0.3 mg were included in the analysis. These consisted of all completed Phase II through IV trials evaluating pegaptanib in AMD available at the time the plan for the pooled analysis was finalized. There were 1,586 subjects enrolled, including 165 (10.4%) with diabetes and 1,421 (89.6%) without diabetes, receiving pegaptanib 0.3 mg. The number of pegaptanib 0.3 mg injections received by the subjects with and without diabetes was similar; the subjects with diabetes received a mean of 8.8 injections (range, 1–39) over a mean of 52.4 weeks and the subjects without diabetes received a mean of 9.7 injections (range, 1–44) over a mean of 57.5 weeks. The diabetic population had a higher percentage of males than the nondiabetic population (51.5% vs. 42.6%) and a lower percentage of Caucasian/white subjects (44.2% vs. 61.3%, respectively); this largely reflected a higher percentage of subjects with an “unspecified” race in the diabetic population (47.9% vs. 31.6%, respectively). The mean age was similar between groups (75.3 [range, 56–89] and 75.9 [range, 40–94] years for the subjects with and without diabetes, respectively).

### Medical history

As expected, the group with diabetes had a greater proportion of subjects with a medical history of metabolic and vascular system disorders, including vascular hypertension, coronary artery disorders, and lipid disorders/analyses; only cardiac arrhythmias and thyroid gland disorders occurred more frequently in subjects without diabetes. Most of the subjects with diabetes (73.3%) and subjects without diabetes (66.1%) reported a history of at least 1 ocular disease in the study eye, with glaucoma and ocular hypertension occurring more frequently in the subjects with diabetes than in those without diabetes (11.5% vs. 7.9%, respectively). Subjects with diabetes also had more eye therapeutic procedures than those without diabetes (21.8% vs. 12.0%, respectively).

### Early discontinuations from treatment

In all, 72 (43.6%) subjects in the diabetic group and 485 (34.8%) subjects in the nondiabetic group discontinued treatment prematurely; causes and their reporting rates were similar between groups. The majority of discontinuations in the diabetic and nondiabetic groups were due to lack of availability of the study drug (31 [18.8%] and 167 [11.8%], respectively) and investigator or sponsor decision (22 [13.3%] and 171 [12%]). There were no deaths leading to early discontinuation in the diabetic group versus 11 (0.8%) in the nondiabetic group. Two (1.2%) subjects in the diabetic group and 16 (1.1%) subjects in the nondiabetic group discontinued due to adverse events.

Adverse events leading to early discontinuation in the diabetic group included 1 subject each with pneumonia not otherwise specified (NOS)/myocardial infarction (unrelated to study drug), and retinal hemorrhage/cerebrovascular accident (causality not specified); both events were fatal. In the nondiabetic group, ocular adverse events leading to discontinuation were macular degeneration including choroidal neovascularization (1 subject), retinal detachment (2 subjects), and visual acuity reduced (3 subjects). Three adverse events resulted in death and early discontinuation: metastatic cancer in 2 subjects and aortic aneurysm rupture in 1 subject; none were related to the study drug. There were no notable differences between the groups in events frequently associated with diabetes; the subjects with diabetes reported 1 occurrence each of myocardial infarction, retinal hemorrhage, and cerebrovascular accident and the subjects without diabetes reported 1 occurrence each of retinal tear and retinal hemorrhage, and 2 occurrences of retinal detachment.

### Treatment-emergent adverse events

Treatment-emergent adverse events (by System Organ Class and PT) were reported by 117 (70.9%) and 1,095 (77.1%) of subjects in the diabetic and nondiabetic groups, respectively, who received pegaptanib. The most common treatment-emergent adverse events were eye disorders, with similar reporting rates in both diabetic and nondiabetic groups (65.5% vs. 66.6%, respectively). In order from highest to lowest, eye pain, reduced visual acuity, punctate keratitis, and vitreous floaters were the most frequent occurrences (reported in 15% to 22% of subjects).

In general, there were no notable differences between groups with respect to treatment-emergent, nonocular adverse events. Among the nonocular adverse events, arthralgia (3.6% vs. 2.4%), chest pain (2.4% vs. 1.2%), dizziness (4.2% vs. 1.4%), aggravated hypertension (3.0% vs. 1.5%), nausea (3.0% vs. 1.8%), and vomiting NOS (4.2% vs. 0.8%) were more frequently reported in subjects with diabetes compared with subjects without diabetes, respectively. Nasopharyngitis was more frequent in subjects without diabetes (4.0%) compared with subjects with diabetes (2.4%).

### Prespecified ocular and hypersensitivity adverse events

In all, 28 (17.0%) subjects in the diabetic group reported 50 prespecified ocular adverse events and 294 (20.7%) in the nondiabetic group reported 601 prespecified events regardless of causality. The incidence and severity of prespecified ocular adverse events (study eye) are shown in Table 3. There were no differences between groups in the incidence or severity of endophthalmitis, retinal detachment, retinal hemorrhage, retinal tear, and vitreous hemorrhage. Retinal pigment epitheliopathy was reported more frequently in subjects with diabetes, and IOP elevations were reported more frequently in the subjects without diabetes. For injection- or therapy-related ocular adverse events, no difference in the incidence or severity of any event was found; 23 injection-related events were reported by 15 (9.1%) subjects with diabetes and 368 events were reported by 164 (11.5%) subjects without diabetes. In all, 9 prespecified ocular events related to study therapy were reported by 4 (2.4%) subjects with diabetes and 57 events were reported by 43 (3.0%) subjects without diabetes. Rash NOS was the only prespecified nonocular (hypersensitivity) adverse event that occurred in both subjects with and without diabetes (1.2% vs. 0.8%, respectively). All other hypersensitivity events were reported in subjects without diabetes (Table 4).

**Table 3:** Incidence and severity of prespecified ocular adverse events in the study eye, all causality

	**Pegaptanib 0.3 mg**					
**Subjects**	**With Diabetes**			**Without Diabetes**		
	**(N = 165 )**			**(N = 1,421)**		
**Preferred Term**^1^	**n**	**(%)**	**Severe**	**n**	**(%)**	**Severe**
Cataract, traumatic	0	0	0	1	(0.1)	0
Detachment of retinal pigment epithelium	0	0	0	3	(0.2)	0
Endophthalmitis	1	(0.6)	1	16	(1.1)	14
Eye hemorrhage	0	0	0	6	(0.4)	2
Eye hemorrhage NOS	0	0	0	3	(0.2)	0
Glaucoma	0	0	0	2	(0.1)	0
Glaucoma NOS	0	0	0	2	(0.1)	0
Intraocular pressure increased	13	(7.9)	0	155	(10.9)	6
Ocular hypertension	0	0	0	11	(0.8)	0
Retinal detachment	1	(0.6)	0	9	(0.6)	5
Retinal hemorrhage	8	(4.8)	1	64	(4.5)	6
Retinal pigment epitheliopathy	4	(2.4)	0	10	(0.7)	0
Retinal tear	1	(0.6)	0	0	(0.4)	0
Vitreous hemorrhage	3	(1.8)	0	21	(1.5)	2

^1^Preferred terms are from MedDRA [*Medical Dictionary for Regulatory Activities, version 12.1*].

NOS: not otherwise specified.

**Table 4:** Incidence and severity of hypersensitivity adverse events, all causality

	**Pegaptanib 0.3 mg**							
**Number of Subjects**	**With Diabetes**				**Without Diabetes**			
	**(N = 165)**				**(N = 1421)**			
**Preferred Term**^1^	**n**	**(%)**	**Severe**	**LT**	**n**	**(%)**	**Severe**	**LT**
Blepharitis, allergic	0	0	0	0	1	(0.1)	0	0
Dermatitis, allergic	0	0	0	0	1	(0.1)	0	0
Dermatitis, contact	0	0	0	0	7	(0.5)	0	0
Drug hypersensitivity	0	0	0	0	6	(0.4)	0	0
Erythema	0	0	0	0	2	(0.1)	0	0
Hypersensitivity	0	0	0	0	2	(0.1)	0	0
Hypersensitivity NOS	0	0	0	0	5	(0.4)	0	0
Rash	0	0	0	0	2	(0.1)	0	0
Rash NOS	2	(1.2)	0	0	12	(0.8)	0	0
Toxic skin eruption	0	0	0	0	1	(0.1)	0	0
Urticaria	0	0	0	0	1	(0.1)	0	0

^1^Preferred terms are from MedDRA [*Medical Dictionary for Regulatory Activities, version 12.1*].

LT: life-threatening.

NOS: not otherwise specified.

### Thromboembolic adverse events

Prespecified APTC adverse events irrespective of causality were reported in 10 (6.1%) subjects with diabetes and in 60 (4.2%) subjects without diabetes. Serious APTC events were reported by 6.1% and 3.4% of subjects with and without diabetes, respectively, and severe APTC events were reported by 3.6% and 2.4%, respectively. Cerebrovascular accident was reported more frequently in the subjects with diabetes than those without diabetes (1.8% vs. 0.6%, respectively), while coronary artery atherosclerosis was only reported in the subjects with diabetes (1.2%; Table 5). No life-threatening events occurred in the subjects with diabetes while 11 (0.7%) were reported in the subjects without diabetes receiving pegaptanib. However, it was noted previously that 2 subjects with diabetes discontinued treatment early: 1 due to myocardial infarction and the other to a cerebrovascular accident; both had fatal outcomes. No prespecified, injection-related APTC adverse events were reported in either group. Cerebrovascular accident, the only study therapy related APTC event, was reported in 1 (0.6%) subject with diabetes and 3 (0.2%) subjects without diabetes.

**Table 5:** **Incidence and severity of prespecified antiplatelet trialists’ collaboration events, all causality**^**1**^

	**Pegaptanib sodium 0.3 mg**								**Sham**			
**Number of Subjects**	**With Diabetes**				**Without Diabetes**				**With Diabetes**			
	**(N = 165)**				**(N = 1421)**				**(N = 26)**			
**Preferred Term**^2^	**n**	**(%)**	**Sev**	**LT**	**n**	**(%)**	**Sev**	**LT**	**n**	**(%)**	**Sev**	**Lt**
Acute myocardial infarction	0	0	0	0	1	(0.1)	0	0	1	(3.8)	0	1
Angina pectoris	1	(0.6)	1	0	8	(0.6)	2	1	1	(3.8)	0	0
Angina unstable	0	0	0	0	4	(0.3)	2	0	0	0	0	0
Cardiac arrest	0	0	0	0	2	(0.1)	0	2	0	0	0	0
Cardiac failure congestive	0	0	0	0	5	(0.4)	1	2	1	(3.8)	1	0
Cerebral hemorrhage	0	0	0	0	1	(0.1)	0	1	0	0	0	0
Cerebrovascular accident	3	(1.8)	3	0	9	(0.6)	5	1	0	0	0	0
Coronary artery atherosclerosis	2	(1.2)	1	0	0	0	0	0	0	0	0	0
Coronary artery disease NOS	1	(0.6)	0	0	9	(0.6)	3	0	0	0	0	0
Coronary artery occlusion	0	0	0	0	1	(0.1)	1	0	0	0	0	0
Myocardial infarction	2	(1.2)	1	0	10	(0.7)	7	2	0	0	0	0
Pulmonary embolism	1	(0.6)	1	0	7	(0.5)	4	1	0	0	0	0
Retroperitoneal hemorrhage	0	0	0	0	1	(0.1)	1	0	1	(0.1)	1	0
Thrombosis	1	(0.6)	1	0	0	0	0	0	0	0	0	0
Transient ischemic attack	1	(0.6)	0	0	8	(0.6)	2	0	0	0	0	0
Silent myocardial infarction	1	(0.6)	0	0	0	0	0	0	0	0	0	0
Ischemia NOS	0	0	0	0	1	(0.1)	0	0	0	0	0	0
Deep vein thrombosis	0	0	0	0	4	(0.3)	1	0	0	0	0	0

^1^In cases in which subjects experienced the same event more than once, the worst severity was presented.

^2^Adverse event with a start date between the day of the first injection and at most 42 days after the last injection.

Preferred terms are from MedDRA [*Medical Dictionary for Regulatory Activities, version 12.1*].

LT: life-threatening.

NOS: not otherwise specified.

Sev: severe.

The analysis identified 26 subjects with diabetes who received sham injection in the 3 controlled studies; baseline demographics were similar to those in the pegaptanib-treated group with diabetes. One APTC event was reported in 3 (11.5%) sham-treated subjects: a life-threatening acute myocardial infarction; moderate severity angina pectoris; severe congestive cardiac failure (Table 5). No events led to death but 1 subject discontinued therapy.

### Deaths

In total, 6 (3.6%) subjects with diabetes and 28 (2.0%) subjects without diabetes died during the studies. Death was considered unrelated to treatment with pegaptanib in 33 cases; in 1 case death due to a ruptured abdominal aortic aneurysm was considered treatment-related. The most common causes of death were attributed to cardiovascular, cerebrovascular or peripheral vascular thrombotic events. Deaths reported in the subjects with diabetes included 1 occurrence of cerebrovascular accident deemed unknown in relationship to study drug administration. Deaths attributed to vascular events in the nondiabetic population included pulmonary embolism (n = 3), myocardial infarction (n = 2), cerebrovascular accident (n = 1), congestive heart failure (n = 2), cardiac/cardiopulmonary arrest (n = 2), ischemic heart disease (n = 2), cardiomyopathy (n = 1), and ruptured abdominal aortic aneurysm (n = 2). The remainder of the deaths were due to polytraumatism, respiratory failure/chronic cardiopulmonary disease, gastric cancer and metastatic colon and prostate cancers, brain metastasis, hypotension, *Clostridium difficile* colitis, chronic obstructive pulmonary disease, bronchopneumonia, and infection complicated by cardiac and renal failure.

## Discussion

In this retrospective examination of pooled adverse events from AMD studies, subjects with diabetes, although more susceptible to disease-related ocular and thromboembolic events, had no increased risk as a result of pegaptanib 0.3 mg therapy compared with those without diabetes. Since only 10% of all subjects had diabetes, comparisons between them and those without diabetes were, at best, unbalanced. Given this limitation, we found no notable differences between the subjects with and without diabetes in the frequencies of events in general or in particular events related to diabetes that were reported. Differences identified between the groups involving adverse events that occurred more often in subjects with diabetes were either expected or were not considered important. The proportions of subjects with diabetes reporting prespecified ocular adverse events were similar to or less than the corresponding proportions reported in the nondiabetic population. The reporting rate and incidence of the prespecified nonocular events (hypersensitivity reactions) were similar between the 2 groups.

The proportions of subjects with diabetes with prespecified APTC adverse events were considered similar to the proportions of those without diabetes with APTC events. However, differences in individual arterial thromboembolic adverse event terms between the 2 populations were identified. Cerebrovascular accident and myocardial infarction were reported with a slightly higher frequency in the subjects with diabetes. There were also 3 events only reported by the diabetic population: coronary artery atherosclerosis, thrombosis, and silent myocardial infarction. In addition, a comparison of nonocular medical histories between the populations indicated that metabolic, vascular, and cardiac disorders were reported more often in the subjects with diabetes, and these disorders were consistent with those generally experienced by a population with a history of diabetes. Based on the deaths reported, there did not appear to be any increased risk of death to subjects with diabetes mellitus as a result of pegaptanib treatment.

Although involving only a small population of people with diabetes receiving sham treatment, these results were further confirmed in the pooled analysis of the 3 studies in which subjects with diabetes were treated with sham injections. Overall, there was no difference in the occurrence of APTC events between subjects with diabetes receiving sham injections and those receiving pegaptanib in the pooled analysis of 9 studies.

As the known properties of VEGF include angiogenesis, increased vascular permeability, and maintenance of vascular endothelial integrity, there are a number of theoretical risks that may be associated with systemic anti-VEGF activity [4]. The systemic administration of high dose bevacizumab in early clinical trials in oncology was associated with potential complications of hypertension, proteinuria, impaired wound healing, gastrointestinal perforation, hemorrhage and thrombosis [23]. The systemic VEGF inhibition effects with pegaptanib are not expected due to VEGF_165_ selectivity and low systemic concentrations following intravitreal administration. In the present study, although the frequency of APTCs was low in both groups, subjects with diabetes appeared to be at an elevated risk of certain APTC adverse events compared to those without diabetes. Thus, the potential for systemic effects following treatment for AMD may be of greater concern in this population. In a 3-year safety study of pegaptanib, there were no thromboembolic cardiovascular accidents or nonocular hemorrhagic events following continuous treatment [18] while in a pooled analysis of data from the ranibizumab trials there was a significantly increased risk of cardiovascular accidents [24] and nonocular hemorrhages [25] in the treated group compared to sham. Bevacizumab has been used increasingly off-label for treatment of AMD [23]; the risk of these side effects following intravitreal injection are not known due to the lack of systematic reporting [25-27]. Of note, in a study of individuals with type 2 diabetes undergoing vitrectomy following intravitreal injection of bevacizumab, mean plasma VEGF concentrations were reduced approximately 10-fold (from 92.0 to 9.7 pg/ml) 1 day after injection, clearly demonstrating entry into the systemic circulation [28].

This retrospective examination of pooled adverse events from AMD studies has certain strengths and limitations. Its strengths include the large multinational population from which the analysis is derived and the rigorous study conduct and monitoring of adverse events in these populations. As noted above, however, the difference in size between the group with diabetes and the group without did not provide a balanced comparison in terms of numbers. Other limitations include combining study protocols that were of differing designs and lengths and pooling clinical studies that were conducted over a period of many years.

## Conclusions

Given consideration of the limitations of the analysis, pegaptanib sodium exposure does not appear to result in an increased risk for arterial thromboembolic or other adverse systemic effects above what would be observed in a sham-treated AMD population with cormorbid diabetes. There does not appear to be an incremental increase in the risk of APTC events in AMD subjects with diabetes due to treatment with pegaptanib.

## Competing interests

All authors are currently and were employees of Pfizer Inc at the time of study conduct and development of the manuscript.

## Authors’ contributions

TD, KK, MS conceived of the work that led to the assembly and review of the acquired data, and the interpretation and submission of the results. TD and MS drafted and revised the manuscript and KK provided a critical review of the content. All authors approved the manuscript for submission.

## Funding

This study was sponsored by Pfizer Inc.

## Pre-publication history

The pre-publication history for this paper can be accessed here:

http://www.biomedcentral.com/1471-2415/12/37/prepub
